# Temporal meal patterns in relation to diet quality and body mass index: findings from a cross-sectional analysis

**DOI:** 10.1007/s00394-025-03857-w

**Published:** 2025-12-04

**Authors:** Jenny Schultz, Anna Karin Lindroos, Ilse Tillman, Lotta Moraeus, Eva Warensjö Lemming

**Affiliations:** 1 Department of Food Studies, Nutrition and Dietetics, Uppsala University, Box 560, 751 22 Uppsala, Sweden; 2 Division for Risk and Benefit Assessment, Swedish Food Agency, Uppsala, Sweden; 3 Department of Food and Nutrition, University of Helsinki, Helsinki, Finland; 4 Medical Epidemiology, Department of Surgical Sciences, Uppsala University, Uppsala, Sweden

**Keywords:** Temporal meal patterns, Chrononutrition, Obesity, Diet quality

## Abstract

**Purpose:**

Although recent studies suggest associations between temporal meal patterns, diet quality, and health outcomes such as obesity and cardiometabolic risk, the evidence remains inconclusive, highlighting the need for further investigation. This study aimed to evaluate meal patterns, including meal frequency, breakfast skipping and timing of energy intake (late or early), and their associations with diet quality and body mass index.

**Methods:**

The study was completed as a secondary analysis of two cross-sectional, national dietary surveys, Riksmaten Adults 2010–11 (n = 1796) and Riksmaten Adolescents 2016–17(n = 2967). Meal patterns were reported for 3–4 days using two different web-based methods. Among adolescents, weight was measured using standardised methods, whereas adults provided self-reported weight. Diet quality was evaluated with the Swedish Healthy Eating Index 2025.

**Results:**

Meal patterns differed according to weight status, where adults with obesity or adolescents with overweight or obesity reported a lower meal frequency and more often skipped breakfast. A low eating frequency, breakfast skipping, and a late energy distribution were negatively associated with diet quality. A high meal frequency (OR 0.44 CI 0.28–0.68) and a late energy distribution (OR 0.70 CI 0.57–0.85) were associated with a decreased risk for overweight or obesity in adolescents.

**Conclusion:**

Our study suggests that there may be a benefit in having a higher eating frequency and consuming breakfast, with regard to diet quality and weight.

**Supplementary Information:**

The online version contains supplementary material available at 10.1007/s00394-025-03857-w.

## Introduction

The relationship between temporal meal patterns, body weight, and cardiometabolic health has gained attention in recent years [1, 2]. Temporal meal patterns describe how food intake is distributed across the day in terms of timing, frequency, and regularity [3] (hereafter referred to as meal patterns). This area of research falls within the concept of chrononutrition, which focuses on how the timing of food intake interacts with the body’s circadian rhythms and thus influence metabolic processes [4, 5]. Evidence suggests that the associations between meal patterns and metabolic health may be caused by the interaction between ingested foods, circadian rhythms, and metabolism, which in turn affects how the body utilises nutrients at different time points of the day and how they influence cardiometabolic functions [5].These potential mechanisms have been described elsewhere [4]. Obesity and diet quality are two other factors that influence cardiometabolic health [6–8], and meal patterns have been associated with both [2, 9].

Given that meal patterns may influence metabolic processes and risk of disease via circadian disruption it is important from a public health perspective to understand their impact on diet quality and obesity. These insights may be used for developing preventive interventions that promote healthier eating habits across diverse populations. Meal patterns, such as meal frequency and lunch- and snack consumption, differ between countries, but few countries provide general recommendations regarding meal patterns, which might be relevant if they are associated with diet- and health-related outcomes [10, 11]. Also, about 15% of the Swedish population between 10 and 80 years skip breakfast regularly, with the highest prevalence in adolescents and younger adults where almost one-third skip breakfast [12]. This has also been shown in other national surveys, such as the US where 20–30% of young adults regularly skip breakfast [13] as well as in observational research where daily breakfast consumption in adolescents ranged between 38 to 73% across Europe and North America [14].

Despite the growing interest, previous research on meal patterns and health-related outcomes has yielded conflicting results, highlighting the need for further investigation in larger, representative populations [15]. Although variables such as meal frequency, breakfast consumption, and late-day food intake have been extensively studied, findings remain inconsistent and methodological heterogeneity and varying definitions complicates direct comparisons across studies [3]. In a previous study, we explored meal patterns in the current study populations and their associations with nutrient intakes, where a high eating frequency and eating breakfast were associated with a higher absolute intake of some key nutrients such as whole grains, vitamin D and folate [12]. Therefore, it is of interest to investigate whether meal patterns are associated with diet quality and risk factors, such as obesity. Moreover, nationally representative data from Sweden on these associations are currently lacking, limiting our understanding of how meal patterns relate to public health in a Swedish context. This study aimed to evaluate the meal patterns variables: meal frequency, breakfast skipping and late versus early energy distribution and their respective association with diet quality, mirrored as the adherence to the Swedish food-based dietary guidelines, and overweight and obesity in two representative samples of the Swedish population. To better understand these relationships, the present study can provide valuable insights for developing public health strategies aimed at improving meal patterns in the population and thus reducing the burden of obesity-related diseases.

## Method

### Population

This study was completed as a secondary analysis of data collected in two cross-sectional, national dietary surveys by the Swedish Food Agency (SFA), Riksmaten Adults 2010–11 (n = 1796) and Riksmaten Adolescents 2016–17(n = 2967). In total, this provided an age span of 10 to 80 years, representative of the Swedish population. The data from the two surveys were analysed separately.

### Riksmaten adolescents

Riksmaten Adolescents 2016–17 was a school-based dietary survey completed in grades 5, 8, and 11 [16]. Statistics Sweden provided a sample of 619 schools to obtain a representative sample. Out of these, 131 schools (22%) agreed to participate, and in total 5145 participants were recruited and invited from one or two chosen classes at each school. Eligible to participate were all students in each class. A total of 3477 (68%) participated in some part of the study, and 2967 (57%) completed all three days of food registrations, which was an inclusion criterion for this study. Participants registered their food for three days using the web-based, validated RiksmatenFlex method, previously described elsewhere [17]. Staff from the SFA visited the schools and guided the participants in how to complete the dietary assessment and measured their height and weight. The first day of the dietary assessment was performed as a 24 h recall and was always the day before the school visit. The second day was a prospective food record of the day of the school visit. The third day was randomly assigned to take place 3–9 days after day 2. All days of the week were represented proportionally. The web-based method is linked to a food composition database specifically adapted for the survey (The Swedish food composition database, version Riksmaten Adolescents 2016–17), which enabled automatic estimation of energy and nutrient intakes. The participants and their parents answered web questionnaires on background characteristics and food intake.

### Riksmaten adults

The Riksmaten Adults 2010–11 survey was a national dietary survey on Swedish adults between 18 and 80 years. Participants were invited to register their food for 4 consecutive days using the web-based, validated Riksmaten method (The Swedish food composition database, version Riksmaten Adults 2010–11) [18, 19], and fill out questionnaires [20]. Further information on the study procedure has been described elsewhere [20, 21]. A representative sample based on age, sex, and living area was drawn by Statistics Sweden. A total of 5003 people were invited, of whom 1796(36%) completed the food diaries. In order to participate, participants needed to speak Swedish. The recruitment was evenly distributed throughout the year. Almost all, 98% of the participants completed all four days, but all participants with approved registrations for at least two days were included in this study. All days of the week were equally distributed in the dataset, as the starting day for each participant was randomly selected.

The food composition database used in the adult survey did not include free sugars. For the present study, intake of free sugars was estimated for adults by utilising a recent version of the Swedish Food composition database (Version 20,240,529). Further, some manual estimation of the free sugars content of some foods was needed since they had been removed in the food database used for the estimation. In total 1908 foods were included in the survey (1699 used) of which 212 foods needed manual estimation of free sugars. Estimation of free sugars was completed with the same method as in the rest of the food composition database [22].

### Background characteristics

Data on age and sex were collected from the class lists for adolescents and for adults, data on age and sex were collected from Statistics Sweden. Age was categorized into the following age groups 18–30, 31–44, 45–64 and 65–80 years for adults, and school grades 5(11–12 years), 8 (14–15 years) and 11(17–18 years) were used for the age categories of adolescents. Physical activity levels were assessed using self-reported questionnaire data. Adolescent participants answered one question about leisure-time physical activity during the previous week. Physical activity level (PAL) for adult participants was computed by combining responses from two questions—one assessing physical activity during work hours and the other evaluating non-work hour related physical activity [23]. Smoking status was collected from the questionnaires (excluding the youngest age group). Education was self-reported in the questionnaires and defined as the participants’ highest education level for adults, and for adolescents the highest education of the parents in the household. In both surveys, some participants provided blood and urine samples, but these were not used in this study.

### Anthropometric measures

Weight and height for adolescents were measured by field staff from the SFA using standardized methods [16]. For adults, weight and height were self-reported in the questionnaires. For adults underweight was defined as body mass index (BMI) < 18.5 kg/m^2^, Normal weight BMI 18.5–24.9 kg/m^2^_,_ Overweight BMI 25.0–29.9 kg/m^2^, and obesity BMI > 30.0 kg/m^2^. ISO-BMI cut-offs weight status were used for adolescents (IOTF) [24]. Associations between meal patterns and obesity in the adult population were explored. Among adolescents, where the obesity prevalence was notably low (4%), relationships between meal patterns and both overweight and obesity were assessed to enhance statistical power.

### Evaluation of energy intake

Under- and overreporting of energy intake were evaluated using the ratio of energy intake (EI) and resting energy expenditure (REE) with the method by Black [25]. REE was calculated based on weight and age [26]. The PAL used in the calculations were the group averages, 1.4 for adolescents and 1.67 for adults. The EI:REE cut-offs was calculated to 0.93–3.01 for adults and 0.83–2.35 for adolescents. Participants outside these cut-offs were identified as misreporters for sensitivity analysis purposes, but were not excluded from the samples.

### Meal patterns

In the web-based dietary assessment, the participants reported their meals using predefined meal types (breakfast, lunch, dinner/evening meal, snack, other eating, drinks only) together with clock time. This information was used to define the participants’ meal patterns. In the present study, we examined meal frequency, breakfast skipping and late energy peak as meal pattern variables. Meal frequency was defined as the number of reported eating occasions per 24 h, with a minimum energy contribution of 50 kcal [3]. Meals only containing drinks were included in this definition. The time between two meals was at least 15 min for adults and one hour for adolescents due to different designs and functionalities of the web-based methods. The mean number of EO was calculated for each participant. Participants were also grouped based on their meal frequency into “3 or fewer meals”, “4 to 5 meals” and “6 or more meals”. Breakfast skipping was identified as participants who did not register the meal-type breakfast on at least one of the days, regardless of clock-time, provided that the breakfast tracked contributed at least 50 kcal. Both irregular breakfast skippers (skipped breakfast on at least one of the registration days) and complete breakfast skippers (no breakfast reported) were grouped into “Breakfast skippers” due to the limited number of cases in each subgroup, which precluded separate analyses.

The distribution of energy during the day was defined as early energy peak when having reported a greater amount of energy between 06.00–14.59 while having reported a greater amount of energy between 15.00–23.59 was called a late energy peak. These cut-offs were used to align with previous studies [9, 12, 27]. The energy intake in each time slot was calculated for all participants and this was calculated to a ratio[12]. A ratio < 1 indicates a late peak and > 1 an early peak. The mean ratio over the reported days per individual was used in the analyses.

### Diet quality index

The Swedish Healthy Eating Index (SHEIA15) was originally developed by Moraeus et al. [28], and has been evaluated for use in both adults and adolescents [28, 29]. The index consists of nine dietary score components based on the Swedish Food-Based Dietary Guidelines (FBDG) 2015 and the Nordic Nutrition Recommendations (NNR) 2012. Since the index was created, both NNR and the dietary guidelines have been updated and the index used in this study has been updated to harmonize with the quantities in the latest Swedish food-based dietary Guidelines of 2025 and NNR 2023 [30, 31]. Thus, the updated index has a new cut-off for red and processed meat intake (< 350 g per week), the recommended intake amount of fruit and vegetables (> 500 g per day, legumes excluded) and the component added sugar was adjusted to free sugar (10 energy percent). This results in the Swedish Healthy Eating Index for Adults or Adolescents 2025 (SHEIA25). The nine components of the index and the calculations are presented in Table 1. Ratios over 1 were adjusted to 1 and ratios below 0 were adjusted to 0; therefore, scores for an individual component could take on any value between 0 and 1. The maximum diet quality score was 9 and a higher score indicates a higher adherence to the FBDG. The dietary index was calculated separately for adults and adolescents.

**Table 1:** Components and calculations of the Diet quality index SHEIA25

Dietary component	Calculation
Fruit and vegetables – at least 500 g per day (potatoes and legumes not included)*	Intake/500
Red and processed meat – max 350 g per week*	1 − ((intake − 350)/350)
Fiber—at least 2.5 g/MJ per day	Intake per MJ /2.5
Whole grain—at least 75 g per 10 MJ	Intake per 10 MJ /75
Fish and shellfish—2 to 3 times per week, average intake 45 g/d	Intake/45
Polyunsaturated fat—at least 7.5 E%	E%/7.5
Monounsaturated fat—at least 15 E%	E%/15
Saturated fat—max 10E%	1 − ((E% − 10)/10)
Added and free sugar—max 10E% *	1 − ((E% − 10)/10)

*Updated since SHEIA15 based on Nordic Nutrition Recommendations 2023 and Swedish food-based dietary guidelines 2025

The dietary data was collected using a method that captures the current intake of individuals. Fish intake is an example of a less frequently consumed food group that can show significant day-to-day variation within individuals [32]. To align with nutritional recommendations, which focus on long-term needs (usual intake), current intake can be converted into usual intake using a statistical method. Multiple methods are available, and for this study the multiple source method (MSM) was used, which has been used in other large studies as well as by SFA [28, 33]. Therefore, before generating the score for fish, the intake of fish and shellfish was converted to the usual intake using the MSM method stratified by sex together with data from the questionnaire on fish consumption [33, 34].

### Statistics

Statistical analyses were performed using STATA version 18 (StataCorp, College Station, Texas, USA). The statistics were performed separately for the two surveys, adolescents and adults, because of differences in data collection procedures and sample size. The significance level was set at *p* < 0.05. Variables were tested for normality with Shapiro–Francia *W*′ test for normality. Differences in SHEIA25 scores, individual SHEIA score components and meal patterns between weight statuses were evaluated using t-test, Mann–Whitney U-test or Pearson’s *x*^2^ test.

A linear regression analysis was performed to explore the association between meal patterns (meal frequency, breakfast skipping and late energy peak) and diet quality. Separate models were performed for each meal pattern variable. A basic model was adjusted for age, and a multivariable model was adjusted for sex, age, physical activity, education level and smoking status (adults). The models were stratified by sex because both meal patterns and diet quality differed by sex. Since the schools could be considered as clusters for the adolescents, a mixed-effect regression model was used with school as a random intercept.

The association between meal patterns and weight was explored using multivariable logistic regression analysis with adjustments for age and sex as model 1 and model 2 further included diet quality, physical activity, smoking (adults) and education level. A mixed-effect logistic model with schools as random intercept were used for adolescents.

Sensitivity analyses were performed by excluding mis-reporters in the multivariable regression analyses on both diet quality and weight status.

## Results

The background characteristics among study participants by survey, school year or age group, and sex are presented in Table 2. A majority of the participants, 56% were women in both populations. The prevalence of obesity was 4% in adolescents and 19% in adults. The prevalence of overweight or obesity in adolescents was 21% and for adults 50%

**Table 2:** Background characteristics and diet quality of study participants in Riksmaten Adolescents 2016–17 and Riksmaten Adults 2010–11

		Adolescents						Adults						
		School year			Sex		All	Age group				Sex		All
		5	8	11	Girls	Boys		18–30	31–44	45–64	65–80	Women	Men	
n		989	1012	966	1656	1311	2967	334	430	665	367	1005	791	1796
± SD		11.5 ± 0.39	14.5 ± 0.40	17.7 ± 0.64	14.6 ± 2.6	14.5 ± 2.6	14.6 ± 2.6	23.7 ± 3.6	37.9 ± 4.0	54.5 ± 5.8	70.4 ± 4.2	47.1 ± 16.7	49.0 ± 16.4	48.0 ± 16.6
Sex n (%)	Men	455(46)	455(45)	401(42)	N/A	N/A	1311(44)	131(39)	184(43)	308(46)	169(46)	N/A	N/A	791(44)
	Women	534(54)	557(55)	565(58)	N/A	N/A	1656(56)	203(61)	247(57)	357(54)	198(54)	N/A	N/A	1005(56)
Weight * status n(%)	Normal weight	676(69)	764(76)	679(71)	1187(72)	932(72)	2119(72)	214(64)	226(53)	269(40)	156(43)	548(55)	317(40)	865(48)
	Overweight	182(19)	141(14)	173(18)	280(17)	216(17)	496(17)	55(17)	115(27)	255(38)	147(40)	255(25)	317(40)	572(32)
	Obesity	35(4)	30(3)	54(6)	58(4)	61(5)	119(4)	55(17)	80(19)	138(21)	61(17)	177(18)	157(20)	334(19)
	Underweight	80(8)	74(7)	54(6)	118(7)	90(7)	208(7)	9(3)	7(2)	3(0)	3(1)	22(2)	0	22(1)
Physical activity** n(%)	1	173(18)	226(22)	303(32)	408(25)	294(23)	702(24)	1.69	1.66	1.66	1.68	1.67	1.67	1.67
	2	222(23)	166(17)	208(22)	387(23)	209(16)	596(20)	N/A	N/A	N/A	N/A	N/A	N/A	N/A
	3	410(42)	365(36)	228(24)	566(34)	437(34)	1003(34)	N/A	N/A	N/A	N/A	N/A	N/A	N/A
	4	173(18)	251(25)	220(23)	288(17)	356(27)	644(21.8)	N/A	N/A	N/A	N/A	N/A	N/A	N/A
Misreporting	Under	69 (7)	100 (10)	92 (10)	145(9)	116(9)	261(9)	74 (22)	48 (11)	106 (16)	45 (12)	146(15)	127(16)	273(15)
	Over	38 (4)	42 (4)	26 (3)	42(3)	63(5)	106(4)	2 (1)	5 (1)	2 (0)	2 (1)	4(0)	7(1)	11(1)
Highest education/parent’s highest education	Primary	41 (4)	48 (5)	35 (4)	67(4)	57(4)	124(6)	17 (5)	15 (4)	91 (14)	136 (37)	124(12)	135(17)	259(14)
	High school	303 (31)	288 (29)	351 (36)	521(31)	421(32)	942(32)	159 (48)	160 (37)	272 (41)	100 (27)	383(38)	308(39)	691(38)
	University	609 (62)	598 (59)	515 (53)	988(60)	734(56)	1722(58)	126 (38)	222 (52)	259 (39)	110 (30)	441(44)	276(35)	717(40)
	None	0	0	0	0	0	0	0	1 (0)	0	2 (1)	2(0)	1(0)	3(0)
	Missing	36 (4)	78 (8)	65 (7)	80(5)	99(8)	179(6)	32 (10)	32 (7)	43 (7)	18 (5)	54(5)	71(9)	125(7)
Smoking	Never	N/A	941 (93)	669 (70)	914(82)	697(82)	1611(82)	199 (66)	266 (67)	286 (46)	155 (44)	508(53)	398(55)	906(54)
	Daily	N/A	0 (0)	50 (5)	33(3)	17(2)	50(3)	26 (9)	26 (7)	66 (11)	26 (7)	88(9)	56(8)	144(9)
	Former	N/A	14 (1)	50 (5)	36(3)	28(3)	64(3)	20 (7)	75 (19)	251 (40)	157 (45)	292(31)	211(29)	503(30)
	Rarely	N/A	29 (3)	168 (18)	115(10)	82(10)	197(10)	56 (19)	32 (8)	20 (3)	15 (4)	67(7)	56(8)	123(7)
	I don’t want to answer	N/A	25 (3)	23 (2)	22(2)	26(3)	48(2)	N/A	N/A	N/A	N/A	N/A	N/A	N/A

*Adults: Underweight BMI < 18.5 kg/m^2^, Normal weight BMI 18.5–24.9 kg/m^2^ Overweight BMI 25.0–29.9 kg/m^2^ Obesity BMI > 30.0 kg/m^2^. Adolescents: ISO-BMI cut-offs, international obesity task force (IOTF) ** Adults: Physical activity level (PAL), estimated from two questions on physical activity at work and at leisure time. Adolescents: Which of the following descriptions fits best on your leisure activities during the last 7 days? 1 = Reading, watching TV or other sedentary activities 2 = Walks, bike rides or other light activities (play, skateboard, rollerblades) for a total of at least 4 h 3 = Participating in exercise sports, e.g. football, swimming etc. or active play, a total of at least 4 h. 4 = Participating in hard training or competitive sports regularly and several times

Misreporting: Participants with EI:REE cut-offs outside cut-offs 0.93–3.01 for adults and 0.83–2.35 for adolescents

Characteristics are stratified by survey, school year or age group, and sex

Meal frequency, breakfast skipping and SHEIA25 score, differed between the adults with obesity and those with normal weight (Tables 3 and 4). Adults with obesity reported a lower meal frequency, were more often breakfast skippers and had lower SHEIA25 score. Same patterns were seen for adolescents, all variables, except diet quality, differed significantly according to weight status with lower meal frequency, a higher proportion of breakfast skippers but a lower proportion of having a late energy peak for those with overweight or obesity compared to normal weight. The mean SHEIA25 score for adults was 6.3 (SD 1.1) of maximum 9 (Table 4), with women having a higher score (6.5 SD 1.1) than men (6.0 SD 1.1) (*p* < 0.001). For adolescents, the mean score was 5.5 (SD 1.0), and girls had a higher score (5.6) than boys (5.4) (*p* < 0.001) (Table 3). There was a difference in the SHEIA25 score between age groups for adults (*p* < 0.001) where SHEIA25 increased with age, but this was not seen among the adolescents (*p* = 0.24) (Supplemental Table 1).

**Table 3:** Diet quality score, Swedish healthy eating index for adults and adolescents 2025 (SHEIA25) and meal patterns by weight status in the adolescent study population

Adolescents						
	Underweight^a^	Normal weight	Overweight	Obesity	All adolescents	*P* value Normal weight vs. overweight or obesity
n	208	2119	496	119	2967	
Meal frequency mean ± sd	4.4 ± 0.9	4.3 ± 0.9	4.0 ± 0.9	3.8 ± 0.9	4.2 ± 0.9	< 0.001
3 or fewer n (%)	40 (19)	459(22)	160 (32)	58(49)	725(24)	< 0.001
4 to 5 n (%)	146 (70)	1526(72)	320 (65)	59(50)	2067(70)	< 0.001
6 or more n (%)	22 (11)	134(6)	16 (3)	2(2)	175(6)	< 0.001
Breakfast skipping n (%)	30(14)	409(19)	112(23)	39(33)	591(20)	< 0.001
Late energy peak n (%)	84(49)	823(39)	156(31)	35(29)	1102(37)	< 0.001
Total SHEIA25^b^ mean ± sd	5.56 ± 1.1	5.53 ± 1.0	5.50 ± 1.0	5.36 ± 1.0	5.52 ± 1.0	0.28
*Individual component score (0 to 1)*						
Fruit and vegetables	0.46 ± 0.27	0.46 ± 0.27	0.42 ± 0.25	0.38 ± 0.22	0.45 ± 0.26	0.014
Meat and processed meat	0.48 ± 0.43	0.46 ± 0.44	0.46 ± 0.43	0.40 ± 0.42	0.46 ± 0.43	0.57
Fiber	0.79 ± 0.19	0.81 ± 0.17	0.79 ± 0.18	0.78 ± 0.18	0.80 ± 0.17	0.38
Whole grains	0.44 ± 0.33	0.44 ± 0.32	0.43 ± 0.33	0.37 ± 0.30	0.44 ± 0.32	0.15
Fish	0.53 ± 0.27	0.51 ± 0.29	0.50 ± 0.30	0.52 ± 0.30	0.51 ± 0.29	0.98
Polyunsaturated fat	0.61 ± 0.15	0.63 ± 0.16	0.64 ± 0.17	0.64 ± 0.17	0.63 ± 0.16	0.75
Monounsaturated fat	0.86 ± 0.12	0.86 ± 0.14	0.87 ± 0.14	0.89 ± 0.13	0.86 ± 0.14	0.07
Saturated fat	0.61 ± 0.25	0.62 ± 0.25	0.62 ± 0.26	0.59 ± 0.26	0.62 ± 0.25	0.82
Added and free sugar	0.77 ± 0.34	0.74 ± 0.34	0.77 ± 0.33	0.78 ± 0.34	0.75 ± 0.33	0.74

^a^Weight status: ISO-BMI cut-offs, international obesity task force (IOTF)

^b^SHEIA25 score can range between 0 to 9 where each of the 9 components can give 0–1 points

**Table 4:** Diet quality score, Swedish healthy eating index for adults and adolescents 2025 (SHEIA25) and meal patterns by weight status in the adult population

Adults						
	Underweight^a^	Normal weight	Overweight	Obesity	All adults	*P* value Normal weight vs. obesity
n	22	865	572	334	1796	
Meal frequency mean ± sd	4.8 ± 1.2	4.7 ± 1.1	4.6 ± 1.0	4.4 ± 1.2	4.6 ± 1.1	< 0.001
3 or fewer n (%)	3 (14)	93 (11)	74 (13)	66 (20)	237 (13)	< 0.001
4 to 5 n (%)	13 (59)	604 (70)	391 (68)	216 (65)	1226 (68)	0.09
6 or more n (%)	6 (27)	168 (19)	107 (19)	52 (16)	333 (19)	0.04
Breakfast skipping n (%)	3 (14)	82 (9)	45 (8)	48 (14)	179 (10)	0.02
Late energy peak n (%)	9 (41)	300 (35)	188 (33)	123 (37)	622 (35)	0.49
Total SHEIA25^b^ mean ± sd	6.19 ± 1.2	6.37 ± 1.1	6.38 ± 1.1	6.02 ± 1.1	6.30 ± 1.1	< 0.001
*Individual component score (0 to 1)*						
Fruit and vegetables	0.57 ± 0.29	0.58 ± 0.28	0.56 ± 0.26	0.49 ± 0.27	0.56 ± 0.27	< 0.001
Meat and processed meat	0.64 ± 0.40	0.52 ± 0.43	0.46 ± 0.43	0.39 ± 0.43	0.48 ± 0.43	< 0.001
Fiber	0.87 ± 0.16	0.88 ± 0.15	0.89 ± 0.15	0.85 ± 0.17	0.88 ± 0.16	< 0.001
Whole grains	0.48 ± 0.34	0.62 ± 0.33	0.62 ± 0.32	0.56 ± 0.32	0.61 ± 0.32	0.006
Fish	0.69 ± 0.30	0.72 ± 0.29	0.72 ± 0.30	0.65 ± 0.31	0.71 ± 0.29	< 0.001
Polyunsaturated fat	0.74 ± 0.17	0.71 ± 0.19	0.72 ± 0.19	0.73 ± 0.19	0.72 ± 0.19	0.12
Monounsaturated fat	0.87 ± 0.14	0.83 ± 0.14	0.83 ± 0.15	0.85 ± 0.15	0.84 ± 0.14	0.002
Saturated fat	0.53 ± 0.34	0.66 ± 0.27	0.69 ± 0.26	0.63 ± 0.27	0.66 ± 0.27	0.11
Added and free sugar	0.79 ± 0.31	0.85 ± 0.26	0.89 ± 0.23	0.86 ± 0.26	0.86 ± 0.25	0.78

^a^Weight status, Underweight: BMI < 18.5 kg/m^2^, Normal weight: BMI 18.5–24.9 kg/m^2^ Overweight: BMI 25.0–29.9 kg/m^2^ Obesity; BMI > 30.0 kg/m^2^

^b^SHEIA25 score can range between 0 to 9 where each of the 9 components can give 0–1 points

The association between meal patterns and SHEIA25 from linear regression models are presented in Table 5. Meal pattern variables were associated with SHEIA25, where a low eating frequency, breakfast skipping, and a late energy peak were negatively associated, while a high eating frequency was positively associated. However, these associations were not statistically significant across all subgroups. Sensitivity analyses, where mis-reporters were excluded, showed that the results were robust.

**Table 5:** Associations (coefficient and 95% Confidence Intervals (CI)) between meal pattern variables and diet quality, Swedish Healthy Eating Index (SHEIA25)

Adolescents					Adults				
		Coefficient	95% CI				Coefficient	95% CI	
*3 or fewer ref. more than 3 EO*									
Girls	Age adjusted	−0.16	−0.28	−0.03	Women	Age adjusted	−0.52	−0.76	−0.29
	Multivariable adjusted	−0.10	−0.22	0.04		Multivariable adjusted	−0.32	−0.56	−0.07
Boys	Age adjusted	−0.14	−0.26	−0.03	Men	Age adjusted	−0.23	−0.42	−0.03
	Multivariable adjusted	−0.10	−0.22	0.02		Multivariable adjusted	−0.14	−0.35	0.06
*6 or more ref. fewer than 6 EO*									
Girls	Age adjusted	0.18	0.02	0.33	Women	Age adjusted	0.19	0.04	0.34
	Multivariable adjusted	0.17	0.01	0.32		Multivariable adjusted	0.14	−0.01	0.29
Boys	Age adjusted	0.06	−0.16	0.27	Men	Age adjusted	0.20	0.02	0.38
	Multivariable adjusted	0.01	−0.20	0.23		Multivariable adjusted	0.15	−0.03	0.33
*Breakfast skipping ref. breakfast eaters*									
Girls	Age adjusted	−0.30	−0.43	−0.18	Women	Age adjusted	−0.63	−0.85	−0.41
	Multivariable adjusted	−0.25	−0.37	−0.13		Multivariable adjusted	−0.50	−0.72	−0.27
Boys	Age adjusted	−0.35	−0.48	−0.22	Men	Age adjusted	−0.33	−0.57	−0.09
	Multivariable adjusted	−0.33	−0.47	−0.20		Multivariable adjusted	−0.26	−0.51	0.00
*Late energy peak ref. early energy peak*									
Girls	Age adjusted	−0.16	−0.25	−0.06	Women	Age adjusted	−0.28	−0.42	−0.15
	Multivariable adjusted	−0.14	−0.24	−0.04		Multivariable adjusted	−0.27	−0.41	−0.14
Boys	Age adjusted	−0.15	−0.25	−0.04	Men	Age adjusted	−0.18	−0.33	−0.03
	Multivariable adjusted	−0.15	−0.25	−0.04		Multivariable adjusted	−0.13	−0.28	0.02

The multivariable adjusted models were adjusted for age, physical activity, education level/parental education and smoking status (adults). For adolescents, the multivariable model was completed as a mixed-effect regression with schools as a random intercept

**Fig. 1:**
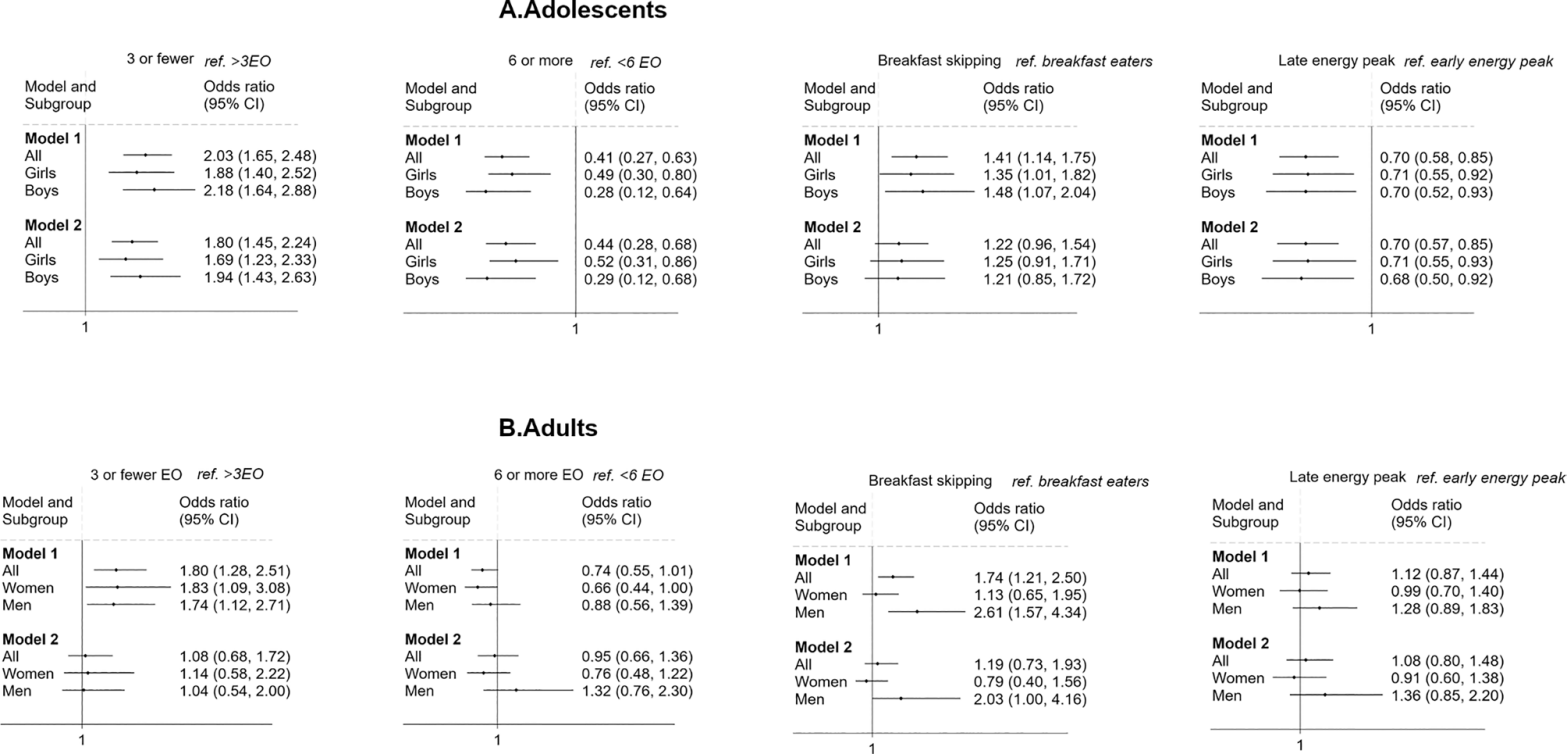
Logistic regression models for the association between meal patterns and obesity (adults) or overweight or obesity (adolescents) are presented as odds ratios. Model 1 was adjusted for age and sex. Model 2 was adjusted for age, sex, diet quality, physical activity, smoking (adults) and education level/parental education. Model 2 was for adolescents completed as a logistic mixed model with schools as random intercept

The association between meal pattern variables and obesity and/or overweight are presented in Fig. 1. The results showed associations between meal patterns and obesity (adults) or overweight or obesity (adolescents) when the models were adjusted for age and sex (Model 1). However, results differed between sexes and between adults and adolescents, where adolescents showed stronger associations than adults did. When models were further adjusted for physical activity, diet quality, smoking habits (adults) and education level (Model 2), there was a higher risk for having obesity in adult men who skipped breakfast (OR 2.03 CI 1.00–4.16) compared to adult men who did not skip breakfast. In adolescents, a low eating frequency was associated with a higher risk for overweight or obesity (OR 1.80 CI 1.45–2.24) while a high eating frequency was associated with a lower risk (OR 0.44 CI 0.28–0.68). Having a late energy peak (more energy consumed late during the day) showed an inverse association with having overweight or obesity in adolescents (OR 0.70 CI 0.57–0.85). In the sensitivity analysis excluding mis-reporters, the association for adolescents with 3 or more EO was attenuated and no longer reached statistical significance.

## Discussion

This study aimed to examine the associations between meal patterns and diet quality as well as meal patterns and overweight and obesity in two representative samples of the Swedish population. The results suggest that temporal meal patterns differ according to weight status in both adolescents and adults. Participants with normal weight had a higher eating frequency and a lower proportion of breakfast skippers, compared to adults with obesity or adolescents with overweight or obesity. The diet quality also differed according to weight status in adults, with those of normal weight having a higher diet quality compared to those with obesity. A low eating frequency, skipping breakfast and having a late energy peak were all inversely associated with diet quality, while a high eating frequency was positively associated in both adolescents and adults. However, the present study found fewer associations between meal patterns and diet quality in the stratified analyses, especially for adult men. Most previous research on adults has also shown positive associations between meal frequency and diet quality [35]. In children and adolescents, cross-sectional studies have shown both positive and negative associations between diet quality and eating frequency as well as with snack frequency [35–37]. Breakfast consumption has, in line with our results, been consistently associated with improved diet quality, and skipping breakfast has been associated with lower diet quality [3, 38–40].

Our results indicate that a higher meal frequency is associated with higher adherence to Swedish dietary guidelines in both adults and adolescent females but not in males. This indicates that the additional meals in males do not improve the diet quality. We did not examine snack frequency per se but meal frequency in this study, and it is likely that these additional meals are snacks. Whether snacks are healthy or not probably differs between countries and individuals, and would affect these relationships. In line with our findings, snacks/between-meal EO has been shown to contribute to the largest proportion of free sugars in this sample of adolescents [41].

Temporal meal patterns were associated with overweight and obesity, especially in adolescents. Taking lifestyle factors such as physical activity, smoking, and education level into account affected these relationships. Skipping breakfast was associated with obesity in adult men (OR 2.03). In adolescents, a high eating frequency (OR 0.44) and a late energy peak (OR 0.70) were associated with a lower risk for overweight or obesity, while a low eating frequency was associated with a higher risk (OR 1.77). In a previous study, we found a positive correlation between the number of meals and energy intake; however, this does not translate into a higher likelihood of overweight and obesity [12]. In both children and adults, most previous cross-sectional studies on meal frequency and body weight have shown a significant inverse association with body mass index(BMI), BMI z-score, and body weight; however, longitudinal research has shown mixed results [35]. Two meta-analyses showed that breakfast skipping was associated with a higher risk of having overweight and obesity, based on cross sectional and cohort studies globally [42, 43]. To gain a deeper understanding of these relationships, study designs beyond cross-sectional approaches are needed.

Misreporting of the energy intake, such as under- and overreporting, also affected the associations between meal patterns and weight status in the present study. This has been reported in previous research as well, and is an important factor to take into account when studying meal patterns [37, 44]. However, excluding these participants may also lead to bias since individuals with obesity tend to under-report their energy intake more [45–47].

A surprising finding in the present study was that in adolescents, a late energy peak was inversely associated with overweight and obesity (OR 0.70). Ingesting more energy later in the day is hypothesized to be less favorable for health due to the metabolic responses of food, such as poorer insulin sensitivity, compared to early in the day [48–50]. A previous meta-analysis has found a positive but small relationship between later eating rhythm and adiposity in children and adolescents, but with low certainty as there were many conflicting results and different definitions of later eating [51]. Previous observational research has shown a higher prevalence of obesity as well as a higher risk for cardiovascular disease risk in adults consuming their last meal later during the day, compared to earlier in the day [1, 52]. However, a meta-analysis of observational studies reported no association between BMI and evening energy intake [53]. A recent study has also evaluated these associations in relation to the polygenic risk of obesity, and found a significant interaction between meal timing and BMI among individuals with high polygenic risk, but not among those with low polygenic risk, suggesting that genetic predisposition should be considered in future research [54].

Adolescents also tend to have a later chronotype, and individuals with evening chronotypes have previously been shown to have higher body weight and being less adherent to a healthy diet [55–57]. The fact that we found an inverse relationship may be explained by that many adolescents who consume more energy later in the day might participate in active leisure activities and therefore eat when they arrive home later in the evening. Still, even though there was a lower risk for obesity, a late energy distribution was associated with a lower diet quality, which also needs to be considered. Foods consumed in the evening are probably of lower nutritional quality.

The large national representative samples and the validated methods used for dietary assessment are main strengths of this study. The combination of the two samples provided an opportunity to evaluate meal patterns in people aged 10–80 years, which is unique. The samples are quite generalizable to the Swedish population, especially for adolescents [16]. While the sampling was representative for adults; the participants were more often highly educated and included a lower proportion of people born abroad [20]. The dietary assessment offered detailed information regarding clock time, meal types and food intake combined.

Several limitations of this study need to be acknowledged. This study is cross-sectional and we cannot conclude any causal relationships between temporal meal patterns, diet quality and overweight and obesity. While we acknowledge that the inclusion of additional covariates such as genetic predispositions, chronic diseases, sleep, or other unmeasured factors could have provided further context, these data were not available in the present study. This study combines two surveys that differ slightly in dietary assessment methods, for example the time between two meals (15 min and 1 h), thus, analyses were carried out separately. Self-reported data can be a weakness, but also minimises external coding errors. Weight and height were self-reported for adults, which can lead to misclassification of weight status.

Knowledge of meal patterns of different population groups, such as people with different weight status, and how meal patterns are associated with diet quality is important information from a public health perspective. Future research is needed to evaluate causal relationships and to evaluate whether there are an association with metabolic health outcomes, especially longitudinal studies. Future research also needs to take misreporting into account when evaluating these relationships based on our findings and other similar research [44]. In addition, future studies could explore how meal patterns interact with circadian systems, energy metabolism, and hormonal factors to better understand the physiological mechanisms underlying these associations.

In conclusion, a low eating frequency, skipping breakfast or having a late energy peak are associated with a lower diet quality. Our study suggests that there may be a benefit in having a higher eating frequency and to consume breakfast, to achieve better adherence to the Swedish dietary guidelines and for weight status.

## Supplementary Information

Below is the link to the electronic supplementary material.

Supplementary file1 (DOCX 19 KB)
