# Supplementary material for: Temporal meal patterns in relation to diet quality and body mass index: findings from a cross-sectional analysis
Source: Eur J Nutr. 2025 Dec 4;65(1):2. doi: 10.1007/s00394-025-03857-w (PMC12678536; doi:10.1007/s00394-025-03857-w)
Supplement: Supplementary file 1 — Supplementary file1 (DOCX 19 KB) [file 394_2025_3857_MOESM1_ESM.docx]

**Temporal Meal Patterns in Relation to Diet Quality and Body Mass Index: Findings from a Cross-Sectional Analysis** Jenny Schultz^1^, Lotta Moraeus^2^, Anna Karin Lindroos^1^, Ilse Tillman^3,^ Eva Warensjö Lemming^1,4^
1. Department of Food Studies, Nutrition and Dietetics, Uppsala University, Sweden
2. Division for Risk and Benefit Assessment, Swedish Food Agency, Uppsala, Sweden
3. Department of Food and Nutrition, University of Helsinki, Finland
4. Medical Epidemiology, Department of Surgical Sciences, Uppsala University, Uppsala, Sweden.

Corresponding author: Jenny Schultz Email: [jenny.schultz@ikv.uu.se](mailto:jenny.schultz@ikv.uu.se)

## Supplemental material

**Table 1**. Swedish healthy eating index 2025 (SHEIA25) and meal patterns of the study population by survey, sex, school year or age group.

|  | **Adolescents** | | | | | | | **Adults** | | | | | | | |  |
| --- | --- | --- | --- | --- | --- | --- | --- | --- | --- | --- | --- | --- | --- | --- | --- | --- |
|  | **Girls** | | | **Boys** | | |  | **Women** | | | | **Men** | | | |  |
|  | **School year** | | | **School year** | | | **All adolescents** | **Age group** | | | | **Age group** | | | | **All adults** |
|  | **5** | **8** | **11** | **5** | **8** | **11** |  | **18-30** | **31-44** | **45-64** | **65-80** | **18-30** | **31-44** | **45-64** | **65-80** |  |
| SHEIA25 score * mean±sd | 5.5 | 5.6 | 5.7 | 5.4 | 5.4 | 5.3 | 5.5±0.02 | 6.1±0.08 | 6.4±0.07 | 6.6±0.05 | 6.9±0.07 | 5.5±0.09 | 5.7±0.08 | 6.1±0.06 | 6.6±0.08 | 6.3 |
| Meal frequency mean±sd | 4.3 | 4.4 | 4.4 | 4.0 | 4.1 | 4.1 | 4.2±0.02 | 4.5±0.07 | 4.8±0.07 | 4.8±0.06 | 4.8±0.07 | 3.9±0.1 | 4.3±0.07 | 4.6±0.06 | 4.8±0.08 | 4.6 |
| 3 or fewer % | 14.4 | 17.1 | 17.4 | 25.1 | 26.1 | 28.9 | 20.9 | 13.8 | 6.5 | 7.8 | 5.6 | 32.8 | 15.3 | 12.7 | 10.1 | 11.7 |
| 6 or more % | 7.9 | 10.8 | 10.6 | 4.0 | 6.2 | 8.48 | 8.16 | 16.8 | 26.7 | 24.4 | 28.8 | 9.2 | 13.7 | 23.7 | 30.8 | 22.6 |
| Breakfast skipping % | 8.8 | 21.9 | 28.0 | 9.5 | 19.6 | 32.9 | 19.9 | 21.2 | 8.9 | 5.6 | 5.6 | 29.0 | 8.2 | 9.1 | 1.2 | 10.0 |
| Late energy peak % | 28.1 | 41.7 | 43.5 | 29.5 | 36.9 | 42.9 | 37.1 | 40.4 | 33.2 | 32.8 | 28.8 | 45.0 | 32.8 | 35.4 | 33.1 | 34.6 |

*SHEIA25 consist of 9 dietary components and each component could give 0-1 points, resulting in total a SHEIA25 of maximum 9.
